# Implementing a new patient navigator model of care within the emergency department for older adults in Ontario, Canada

**DOI:** 10.1371/journal.pone.0315641

**Published:** 2025-03-06

**Authors:** Grace Liu, Amanda Knoepfli, Tracey DasGupta, Naomi Ziegler, Emma Elliot, Mahala English, Sander L. Hitzig, Sara J.T. Guilcher

**Affiliations:** 1 John’s Rehab Research Program, Sunnybrook Research Institute, Sunnybrook Health Sciences Centre, Toronto, Ontario, Canada; 2 SPRINT Senior Care, Toronto, Ontario, Canada; 3 Sunnybrook Health Sciences Centre, Toronto, Ontario, Canada; 4 Lawrence S. Bloomberg Faculty of Nursing, University of Toronto, Toronto, Ontario, Canada; 5 Rehabilitation Sciences Institute, Temerty Faculty of Medicine, University of Toronto, Toronto, Ontario, Canada; 6 Department of Occupational Science and Occupational Therapy, Temerty Faculty of Medicine, University of Toronto, Toronto, Ontario, Canada; 7 Leslie Dan Faculty of Pharmacy, University of Toronto, Toronto, Ontario, Canada; 8 Department of Physical Therapy, Temerty Faculty of Medicine, University of Toronto, Toronto, Ontario, Canada; Mayo Clinic College of Medicine and Science, UNITED STATES OF AMERICA

## Abstract

A Patient Navigator (PN) role was introduced in the Emergency Department (ED) in a large metropolitan hospital in Southern Ontario (Canada) to assist with care transitions. The purpose of this study was to describe the new PN program and type of services provided for older adults in the ED. Given the novelty of the program, it is critical to better understand how a PN ED model of care may help improve the discharge process and ED-community transitions for older adults. This retrospective observational cohort study includes data between November 2020 and October 2021. In this study, the clinical data collected by the PN were analyzed to describe the patient socio-demographics, types of services provided, and outcomes. The PN contacted 95% patients (n = 125) referred to the service in which the median age was 80 (SD = 9.0) consisting of mostly females (74%; n = 92). The PN provided consultations to 79 patients (≤7 days) and 46 patients were admitted to the PN’s caseload. For the 46 admitted cases, the PN connected to 52% of the patients on the same day, facilitated 83% of the patients in returning home or supportive setting and provided follow-up care (i.e., phone calls or home visits) for 67 days (median) in the community. This study provides a preliminary depiction of the scope of practice of a PN within an ED setting, and important considerations for decision-makers and/or administrators interested in implementing a PN role in the ED.

## Introduction

There is an Emergency department (ED) crisis globally [1]. ED visits are meant to provide urgent care for acute health conditions, but more people, including in Canada, are using the ED for non-emergent reasons (i.e., not an acute injury or illness that poses an immediate risk to a person’s life) [2]. In addition, some of the repeat ED visits may perhaps be compounded by unmet needs due to a lack of primary care and/or social care services available to effectively support older adults [1]. For older adults, the transition from hospital to home or from the ED to the community can often be challenging for them and their families/caregivers [3], particularly for patients with complex health and social needs [4]. Many older adults discharged from the ED often expressed frustration over their abilities to perform activities of daily living and/or concerns about being discharged due to lack of being involved or may not have received needed information [5]. Although there are various interventions and strategies in the literature to improve transitional care from hospital to home, effective transitional care models have not been established for the older adult population [6].

Several systematic reviews on discharging older patients from the ED [3,7,8] were unable to identify evidence for effective care models. A contributing factor affecting the development of effective care models may be the need for greater clarity and consensus by emergency clinicians to determine the most appropriate discharge measures, screening tools, information sources and discharge roles for the ED [8]. This includes providing an interdisciplinary assessment of the patient’s level of frailty that involves a thoughtful review of their medications, a screen for other geriatric syndromes, and a care plan that addresses the individual’s needs, which takes into account the patient’s goals and preferences once discharged from the ED [3,9]. Other possible interventions or approaches includes involving caregivers, providing management education, coaching, home visits, and telephone call visits [3]. Relatedly, there is a need for data to inform optimal duration of potential post-ED discharge approaches [10].

Although specific evidence is generally lacking, the Canadian Association of Emergency Physicians recommends that multiple interventions be implemented [11], including care coordination, obtaining collateral information from various sources, coordinating transitions of care, providing community follow-up, and accessing support services which can be done by a geriatric nurse, social worker or another trained clinician [12]. There is evidence to suggest that interdisciplinary care of older adults leads to reduced ED length of stay, decreased ED revisits, decreased hospital admissions, improved functional outcomes, and increased system-level healthcare cost savings [13].

An emerging mechanism that may serve to effectively support ED transitions for older adults are patient navigator (PN) programs [14]. PN programs enable timely access to healthcare services, support hospital-to-home transitions, and provide follow-up care [15–17]. In this paper, a PN model of care is defined as a person-centred healthcare service focused on creating a seamless flow for patients as the journey through the care continuum [18]. The PN engages with the patients and/or their families/caregivers to identify barriers to accessing care, provide referrals to services, facilitate transitions of care and promote self-management [16,19]. Since there are different roles, functions, background and models of care of PN programs, further research is needed regarding the most appropriate navigator for specific populations [20].

There is a need for quantitative evidence to characterize the types of patients being referred to the PN program, including the delivery method best suited for a given setting and program outcomes [20]. Further guidance on best practices for implementation of PN programs is needed including the duration and frequency of a PN [20]. Since this innovative model of care is relatively new, further research is needed to demonstrate the impact of PN programs for all populations and to explore PN caseloads and/or cost-effectiveness across the disease continuum in a variety of healthcare settings [20]. Within the ED context, a PN may lead to a reduction in avoidable ED follow-up visits and increase in follow-up appointment adherence [21], but further work is required to obtain data to demonstrate if this is possible.

At our clinical site, which is a major acute trauma hospital in Southern Ontario (Canada), a PN program was developed and implemented hospital wide. The PN is a social worker (Community Transitional Worker) that is employed by a partnering community agency who is embedded as part of the hospital’s teams to support older adult patients with complex care needs. Any physician, hospital or community providers, or patients/family caregivers can make a referral to the PN program. Upon referral, the PN would assess the patients’ needs and provide follow-up care for up to 90 days (or long as needed), which may include a home visit.

Qualitative findings from an earlier iteration of the PN program at our site found that older adults and their caregivers noted high rates of satisfaction with the PN program [22]. However, there is a need to also examine quantitative data to better understand how this novel care model may help to improve the discharge process and ED-community transitions for older adults. Hence, the current study evaluated a new PN model of care within the hospital’s ED to support the older adult population by obtaining metrics that could serve to inform the scope of practice by PNs in the ED, and to describe patient discharge outcomes.

## Methods

### Study design

The present study used a retrospective cohort observational design guided by the ‘Strengthening the Reporting of Observational Studies in Epidemiology’ (STROBE) statement to track a group of patients on the PN’s caseload who works in the hospital’s ED. (see S1 File: STROBE) [23]. We extracted data by reviewing the patient tracking sheet, which is a standard clinical tool used with all patients receiving routine care by the PN. As per our hospital’s policies and research ethics board guidelines, routinely collected clinical data deemed as secondary data are allowed to be used for research purposes. As such, informed consent was waived for reviewing the patient tracking sheet. No data specific for research purposes were collected (e.g., beyond standard care) and all personal health information was fully anonymized. Ethics approval for this study was obtained from the research ethics boards at the Sunnybrook [REB 1598] and SPRINT Senior Care.

### Setting/participants

The inclusion criteria were older adults referred to the PN program in the hospital’s ED (from November 2020 to October 2021). For the data analysis, we included patients who were contacted by the PN for the first-time (age ≥  60 years). Patients who were followed for ≤  7 days were categorized as consultations since they were not formally added to the PN’s caseload. Consultations are characterized as the initial PN contact whereby a patient’s suitability for the PN program is assessed or where only a brief interaction was required (e.g., to provide information about available services) with no sustained follow-up. Patients admitted to the PN caseload received follow-up care determined by the PN, which may include a home visit.

### Data collection & analysis

The data collected from the patient tracking sheet included: 1) patient socio-demographics profile (i.e., chronological age, sex), 2) PN services provided (i.e., types of interventions, response time, duration, and if there was a home visit); and 3) program discharge location. See Table 1 for a list of our Variable Definitions and Statistical Analysis.

**Table 1:** Variable definitions and statistical analysis.

Data	Variable Definition	Statistical Analysis
**Age**	Quantitative variable (in years)	Mean, Median, Range, SD, Interquartile Range, Unknown
**Sex**	Nominal variable (Mutually exclusive) 1) Female 2) Male 3) Unknown	Count, %
**Response Time**	Calculation: Days from Referral Date to PN Initial Contact Date	Mean, Median, Range, SD, Interquartile Range, Count, %
**Program Duration**	Calculation: Days from PN Initial Contact Date to PN Program Discharge Date	Mean, Median, Range, SD, Interquartile Range, Count, %
**Interventions**	Nominal variable (Not mutually exclusive) 1) Service Connection 2) Discharge Planning 3) Provider Connection 4) Social Support 5) Instrumental Activities of Daily Living 6) Housing Alternatives 7) Caregiver Support	Count, %
**Post-Discharge Location**	Nominal variable (Mutually exclusive) 1) Home or to a supportive setting, 2) Acute, Rehabilitation, Transitional Care or Long-Term Care 3) Other: Includes cases which were unknown, shelter, or deceased	Count, %

Note: Data source extracted from PN standard clinical tool. Descriptive statistics were used to analyze the data. Sample size and power calculations were not considered in this analysis.

Data were screened for completeness and reviewed by the first author (GL) and the co-senior-responsible author (SLH). For classifying the PN interventions, we used a similar classification method to our previous study [24]. The themes included: Service connection, Discharge planning, Provider connection, Social support, Instrumental activities of daily living, Housing alternatives and Caregiver support. Open-ended descriptive detailing types of interventions were coded by another member of the team (ME—undergraduate student), which were reviewed by the first author (GL—Post-doctoral fellow). Frequencies and descriptive statistics were used to analyze the data. The summary results were presented and validated by the PN management team (AK, NZ).

## Results

### Referrals

Of the 147 cases referred to the PN program in the hospital’s ED between November 2020 and October 2021, 7 patients were unable to be contacted, and 15 did not require or declined PN services. Based on the 125 cases contacted, 79 patients received consults (i.e., service ≤  7 days), which were referred to as the ‘PN consultation group’, and 46 were admitted to the PN caseload, which were referred to as the ‘PN caseload group’.

### Socio-demographics

Of the 125 patients contacted, the mean and median age was 80 (SD = 9.0), ranging from 60 to 104 years (IQR = 12.3). Age was missing for five cases. The majority of the sample was female (74%; n = 92 vs. 25% male; n = 31), with 2% (n = 2) having missing data. In the PN consultation group, the mean and median age were 81 (SD = 9.0; IQR = 13.0; range 62 to 104) with 76% females. In the PN caseload group, the mean and median age was 79 (SD = 9.0; IQR = 14.0; range 60 to 98) with 70% females. The socio-demographics profiles (chronological age, sex) are displayed in Table 2.

**Table 2:** Patient navigator ED patients, response times, and duration.

	Total Patients Contacted (N = 125)	PN Consultation Group (n = 79)	PN Caseload Group (n = 46)
**Age (years)**			
Mean	80	81	79
Median	80	81	79
Range	60–104	62–104	60–98
SD	9.0	9.0	9.0
IQR	12.3	13.0	14.0
Unknown	5	4	1
**Sex (count,%)**			
Females	92 (74%)	60 (76%)	32 (70%)
Males	31 (25%)	18 (23%)	13 (28%)
Unknown	2 (2%)	1 (1%)	1 (2%)
**Response Time (days)**			
Mean	2	2	3
Median	0	0	0
Range	0–21	0–12	0–21
SD	3.6	2.8	4.5
IQR	3.3	3.0	4.0
**Response Time (count, %)**			
Same day	64 (51%)	40 (51%)	24 (52%)
Within 7 days	42 (34%)	24 (30%)	18 (39%)
From 8–14 days	5 (4%)	3 (4%)	2 (4%)
≥ 15 days	2 (2)%	0 (0%)	2 (4%)
Missing	12 (10%)	12 (15%)	0 (0%)
**Program Duration (days)**			
Mean	47	1	92
Median	9	0	67
Range	0–350	0–7	10–350
SD	70.3	2.2	76.1
IQR	60	1.5	124.5
**Program Duration (count, %)**			
Within 7 days	43 (34%)	43 (54%)	0 (0%)
From 8–30 days	12 (10%)	–	12 (26%)
From 31–60 days	9 (7%)	–	9 (20%)
From 61–90 days	3 (2%)	–	3 (7%)
From 91–180 days	14 (11%)	–	14 (30%)
From 181–270 days	4 (3%)	–	4 (9%)
From 271–360 days	1 (1%)	–	1 (2%)
Ongoing	3 (2%)	–	3 (7%)
Missing	36 (29%)	36 (46%)	0 (0%)

### PN response time

Upon receipt of the referral, the PN response time to meeting with the patient and/or caregivers was on average within two days (SD = 3.6, Median = 0 days, IQR = 3.3) (see Table 2), which ranged from 0 to 21 days. The response time was as follows: 51% (n = 64) same day, 34% (n = 42) within 7 days, 4% (n = 5) from 8 to 14 days, and 2% (n = 2) from 15 days or greater. In some of the cases, the patients were discharged home before they were seen by the PN in the ED, in which the PN would provide follow-up care over the phone or may conduct a home visit (if needed). Data were missing for 12 (10%) of the cases. The PN response times are displayed in Table 2.

### PN program duration

For the 79 patients (63%) in the PN consultation group (Table 2), the PN program duration was on average 1 day (SD = 2.2, Median = 0 days, IQR = 1.5) ranging from 0 to 7 days. Data were missing for 36 cases (46%). For the 46 patients (37%) in the PN caseload group, the PN program duration was on average 92 days (SD = 76.1, Median = 67 days, IQR = 124.5) ranging from 10 to 350 days. Overall, program duration were as followed: 34% (n = 43) from 0 to 7 days, 10% (n = 12) from 8 to 30 days, 7% (n = 9) from 31 to 60 days, 2% (n = 3) from 61 to 90 days, 11% (n = 14) from 91 to 180 days, 3% (n = 4) from 181 to 270 days, and 1% (n = 1) from 271 to 360 days. For three (2%) of cases, the patients required ongoing support, and did not have a defined discharge date. The PN program durations are displayed in Table 2.

### PN interventions

For the 79 patients in the PN consultation group, they received the following interventions: service connection to community programs/services (85%, n = 67), discharge planning (49%, n = 39) and provider connection (i.e., physicians, rehabilitation, mental health) (34%, n = 27). The PN provided counselling support to patients who were socially isolated or emotionally distressed (22%, n = 17) and caregivers who were stressed or burnout (9%, n = 7). The PN provided guidance with instrumental activities of daily living (i.e., link to homemaking, grocery delivery, and home maintenance services; guide in scheduling follow-up appointments and set up personal emergency response systems) (17%, n = 13). The PN also discussed housing alternatives and provided support to the patients and their families/caregivers while the patient was transitioning to another facility (i.e., retirement home, supportive housing, long-term care) (20%, n = 16). The PN interventions are displayed in Table 3. 

**Table 3:** Interventions provided by the patient navigator.

PN Interventions	Total Patients Contacted (N = 125) (%)	PN Consultation Group (n = 79) (%)	PN Caseload Group (n = 46) (%)
**Service Connection** (e.g., connect to community programs and services such as personal support worker, transportation, etc.)	104 (83%)	67 (85%)	37 (80%)
**Discharge Planning** (e.g., plan/support care while transitioning from hospital to home, equipment needs, coordination, etc.)	82 (66%)	39 (49%)	43 (94%)
**Provider Connection** (e.g., connect to physician, allied health professionals, or other clinical services such as mental health and addiction, etc.)	44 (35%)	27 (34%)	17 (37%)
**Social Support** (e.g., counsel patients who live alone or are socially isolated, provide support due to emotional distress or risk of abuse, etc.)	28 (22%)	17 (22%)	11 (24%)
**Instrumental Activities of Daily Living** (e.g., link to services such as homemaking, grocery delivery, and home maintenance; guide patients/families in managing their care needs such as scheduling follow-up appointments and setting up personal emergency response systems, etc.)	27 (22%)	13 (17%)	14 (30%)
**Housing Alternatives** (e.g., discuss housing alternatives and options, provide support while transitioning to another facility such as retirement home, supportive housing, Long-Term Care, etc.)	20 (16%)	16 (20%)	4 (9%)
**Caregiver Support** (e.g., support family and/or caregivers who are stressed, at risk of burnout or experiencing burnout, etc.)	19 (15%)	7 (9%)	12 (26%)

For the 46 patients in the PN caseload group, they received the following interventions: service connection to community programs/services (80%, n = 37), discharge planning (94%, n = 43) and provider connection (37%, n = 17). The PN provided counselling support to patients who were socially isolated or emotionally distressed (24%, n = 11) and caregivers who were stressed or burnout (26%, n = 12). The PN provided guidance with instrumental activities of daily living (30%, n = 14). The PN also discussed housing alternatives and provided support while transitioning to another facility (9%, n = 4). Of the 46 patients in the PN caseload group, seven home visits were conducted (15%). The PN interventions are displayed in Table 3.

### Post-discharge location

The post-discharge location for most patients (both PN consultation and PN caseload groups; 72%, n = 125) was home or to a supportive setting. For the 79 patients in the PN consultation group, the post-discharge location for most patients (66%, n = 52) was home or to a supportive setting. The latter (25%, n = 20) were admitted to other settings (i.e., acute care, inpatient rehabilitation, transitional care or long-term care). For seven patients (9%), the discharged location was ‘Other’ (which were unknown, shelter, or deceased). Out of the 46 patients in the PN caseload group, the PN facilitated 83% (n = 38) of the patients in returning home or to a supportive setting. The latter (9%, n = 4) were admitted to other settings. For four patients (9%), the discharged location was ‘Other’ (which were unknown, shelter, or deceased). The post-discharge location are displayed in Table 4. 

**Table 4.** Post-discharge location of patients from patient navigator program.

Post-Discharge Location	Total Patients Contacted (N = 125) (%)	PN Consultation Group (n = 79) (%)	PN Caseload Group (n = 46) (%)
Home or to a supportive setting	90 (72%)	52 (66%)	38 (83%)
Acute, Rehabilitation, Transitional Care or Long-Term Care	24 (19%)	20 (25%)	4 (9%)
Other: Includes cases which were unknown, shelter, or deceased	11 (8%)	7 (9%)	4 (9%)

## Discussion

The purpose of this study was to describe the types of patients being supported by the PN program, services provided, and their discharge outcomes. In this retrospective observational cohort study, the PN supported more females (74%) compared to males. However, we did not compare the proportion of females and males in the ED to our sample population. Based on our data, the PN seemed to help with the discharge process to triage the majority of patients (72%) to a home or supportive setting. The remaining patients were discharged to other care settings, which in itself may indicate that patients were referred to appropriate care settings to meet their respective needs. Older adults can often be discharged home if they receive social and nursing support [25]. Since discharge decisions require assessment and identification of medical, functional, and social issues for older adults with complex needs [25], the PN may have facilitated the discharge process by perhaps helping to determine the patients’ specific needs and assisting them while transitioning back to their home. Qualitative data on a similar PN role at our site, which included supporting patients within the ED, provides some corroboration for this possibility [4,22].

In terms of supports, the PN provided various interventions to support older adults and their caregivers, which included service and provider connection, social and caregiver support, instrumental activities of daily living, and housing alternatives. In particular, the embedded PN in the ED appeared to facilitate appropriate referrals based on the high proportion returned to a home or home setting. This type of knowledge is critical since older adults often are unaware of community services available to them and encounter barriers in accessing adequate in-home care or to healthcare and social care providers [11]. Further, there is evidence showing that effective collaborations between health and community care are essential for patients with complex care needs [26]. As such, it is conceivable that the embedded PN from a community-based organization within the hospital setting played an important role in linking patients and caregivers to appropriate health and social care services.

Notably, approximately 22% of patients were socially isolated or emotionally distressed while almost 15% of caregivers were stressed or reporting feelings of being “burnout” to the PN. Hence, the PN’s scope of practice addressed these needs, which can be critical for maintaining the wellbeing of patients and their family members [10,17,27]. For older adults experiencing social isolation, there are significant physical and mental health issues that can arise from being socially disconnected [28], which can lead to further adverse events if not addressed.

The PN was involved in providing information to the patients about follow-up care (i.e., further examinations or treatments) to help older adults be more prepared for discharge [5]. When reviewing the types of services provided by the PN, the role appeared to have some overlap with the role of social workers, discharge planners, case managers in the community, as well as a primary care provider since they prepared the patients and their care partners for discharge, and then provided them with ongoing follow-up care. Based on input from the PN service provider, we know that if the patient already had a case manager or primary care provider in the community, the PN in the ED would liaise with them to provide a warm transfer. However, for patients who do not have a case manager, the PN provided guidance on several instrumental activities of daily living (i.e., link to homemaking, grocery delivery and home maintenance services; schedule follow-up appointments) to help older adults and their care partners even after they have been discharged from the hospital ED.

Based on an earlier iteration of the PN program at our site, the key components of an ‘ideal’ PN program for older adults are: (1) Easy accessibility and open communication; (2) flexible eligibility requirements; (3) characteristics of the PN; and (4) appropriate program size and duration [14]. The data in our study supports that the PN may have had many of these qualities since the PN appeared to be able to rapidly respond to patient needs. For instance, the PN was able to meet with approximately 50% of patients quickly within the same day. Timely access is critical to support older adults in the ED given that older adult patients presenting to the ED often experience challenges with ED-community transitions due to the short ED visit and quick turnaround time [5]. However, the PN only worked full-time Monday through Fridays during regular hours to support patients and their families/caregivers. Hence, further work is required to determine barriers and facilitators to timely PN access to patients in the ED and to those admitted to hospital to determine if there are factors that could accelerate this process.

**Fig 1:**
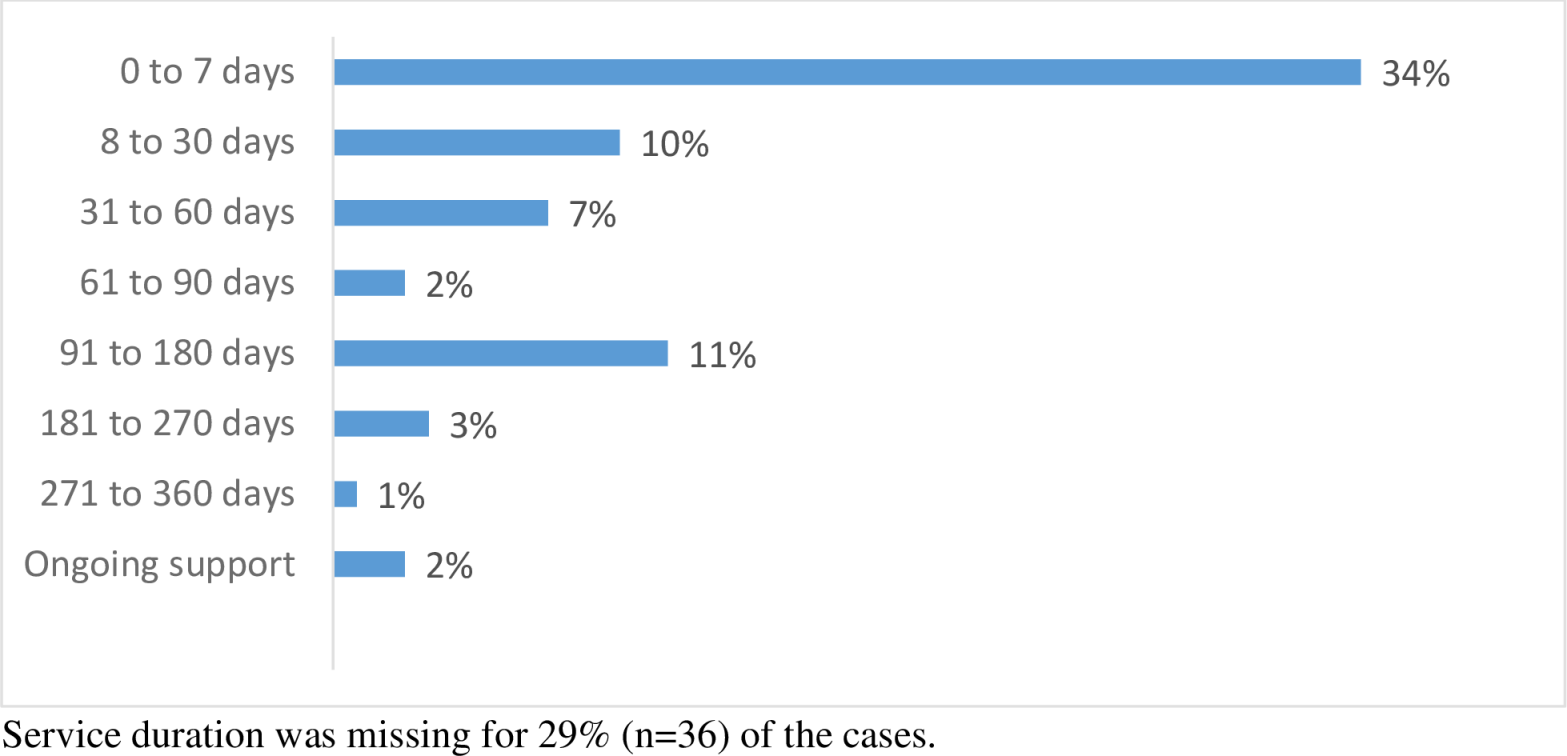
Service duration provided by the patient navigator.

For the PN caseload group, the service duration varied from 10 to 350 days, and the PN provided ongoing follow-up care in a few cases (Fig 1). Hence, there were some cases where the pre-determined boundary of 90-day follow-up was not sufficient for a small portion of patients, which may have been attributed to the complexity of their needs. Currently, there is no evidence for the recommended service length for PN programs [29] but it appears that the 90-day follow-up was sufficient for the majority, with most being discharged by the PN within that timeframe. Because the service duration was missing and reason(s) for discharge was not clearly indicated in some of the PN’s clinical notes, the 90-day follow-up period cannot be generalized. Additionally, the PN program was set up to allow patients/families to be followed-up for as long as needed.

### Study strengths & limitations

This was a retrospective observational cohort study design with no controls and limited data collected within a one year period (between November 2020 and October 2021). However, this study provides important insights regarding a novel PN program for older adults in a hospital’s ED where the patients are followed-up by a social worker or ‘community transitional worker’. Given that a standardized PN clinical tool was not established for the ED, we developed a tool to capture data on patients socio-demographics (i.e., age and sex), PN services (i.e., response time, duration, and interventions), and program discharge location. Although we did not collect patient-reported outcomes (i.e., quality of life) in this study; in an earlier iteration of the PN program at our site, we found that older adults and their caregivers noted high rates of satisfaction with the PN program [21].

The data from the clinical tool were subsquently mobilized through our use of an implementation science approach to engage with hospital providers and community care partners to support their ability to collaborate and respond to the needs of older adult patients and their families/caregivers [21]. Specifically, our research team provided data summaries of our findings to both hospital and community decision-makers to inform PN implementation and to facilitate integrated care [30]. We recommend that facilities interested in implementing a PN program use an implementation science approach to inform their evaluation of the services to optimize outcomes and ensure sustainability. 

In terms of other limitations, we did not capture relevant contextual factors related to PN referral. For example, while staff in the ED were encouraged to refer their patients to the PN program for follow-up care, we did not consider the number of referrals to the PN program in comparison to the ED volumes during the same period of time. Also, we did not capture the PN’s workload on a day-to-day basis to track the duration and frequency the PN was involved consulting with the ED team and assessing the patients’ needs in the hospital ED, compared to the time and number of follow-up phone calls or home visits conducted. 

There were also missing data in the PN clinical notes, including socio-demographics, response time, service duration, interventions provided, and post-discharge location. As well, the reason for admission to the ED, patients’ diagnosis or socioeconomic status and if the older adult patient had any family/caregiver support were not consistently documented. Since this study only provides a descriptive account of the PN program and lacked a comparison cohort group (older adult patients not seen by a PN), most of the analysis was limited to the use of counts, percentages and other descriptive statistics (i.e., mean, median, range, standard deviation and interquartile range). 

Key performance metrics related to ED visits were also not captured in the data, such as length of time in the ED. Although the PN captured information on patients who may have had multiple ED visits in the clinical notes, we were unable to determine the exact number of repeated ED readmissions. It would be valuable to determine whether the PN program in the ED can decrease future unnecessary ED visits and hospital readmissions since this could be a sign that health care and post-discharge care are not meeting the needs of older adults [1]. Overall, future studies on ED PN services should follow participants over a longer period of time, and collect: a) data on the reason(s) for ED admission and number of ED readmissions; b) additional data related to patients’ characteristics and patient-reported outcomes, and; c) more details regarding the PN's workload. Relatedly, this should include undertaking an analysis of other providers whose roles and responsibilities overlaps with the services by the PN (i.e., social workers, discharge planners, case managers, etc.) to better understand the unique contributions of this role in patient care.

Despite these limitations, this study contributes to the research on implementing a PN model of care in a hospital’s ED by providing some insights about the types of patients who received care by an ED PN, the types of services being delivered by the PN, and where patients were discharged to post-hospital visit. At this time, there are several gaps in our understanding of what constitutes PN for older adults [31], and the data from this study can serve to inform other sites interested in developing their own PN programs and illustrating some potential key metrics of PN performance (i.e., response time and service duration).

## Conclusion

This study describes a novel PN program for older adults in a hospital’s ED where patients are seen and followed-up by a ‘community transitional worker’. Although our findings are preliminary and descriptive in nature, it appears that PN services in the ED setting can help older adults with complex care needs receive appropriate and timely supports to assist them in returning to a home setting. This study provides a depiction of the scope of practice of a PN within an ED setting, which may be useful for decision-makers and administrators interested in implementing a PN role in the ED and help support older adult with transitions of care.

## Supporting information

S1 File “Strengthening the Reporting of Observational Studies in Epidemiology” (STROBE). (DOCX)
